# FdeC expression regulates motility and adhesion of the avian pathogenic *Escherichia coli* strain IMT5155

**DOI:** 10.1186/s13567-024-01327-5

**Published:** 2024-05-31

**Authors:** Adrianna Aleksandrowicz, Rikke Brødsgaard Kjærup, Krzysztof Grzymajło, Fernando Garcia Martinez, Javier Muñoz, Dominika Borowska, Samantha Sives, Lonneke Vervelde, Tina Sørensen Dalgaard, Robert A. Kingsley, Rafał Kolenda

**Affiliations:** 1 Department of Biochemistry and Molecular Biology, Faculty of Veterinary Medicine, Wrocław University of Environmental and Life Sciences, Wrocław, Poland; 2 Department of Animal and Veterinary Sciences, Aarhus University, Aarhus, Denmark; 3 Proteomics Unit, Spanish National Cancer Research Centre (CNIO), Madrid, Spain; 4 Present Address: Cell Signaling and Clinical Proteomics Group, Biobizkaia Health Research Institute, Barakaldo, Spain; 5 Present Address: Ikerbasque, Basque Foundation for Science, Bilbao, Spain; 6 Division of Immunology, The Roslin Institute and Royal (Dick), School of Veterinary Studies, University of Edinburgh, Edinburgh, UK; 7 Quadram Institute Biosciences, Norwich Research Park, Norwich, UK; 8 University of East Anglia, Norwich, UK

**Keywords:** *Escherichia coli*, adhesion, FdeC, adhesin, autotransporter, motility, regulation, APEC, proteomics

## Abstract

Adaptation of avian pathogenic *E. coli* (APEC) to changing host environments including virulence factors expression is vital for disease progression. FdeC is an autotransporter adhesin that plays a role in uropathogenic *Escherichia coli* (UPEC) adhesion to epithelial cells. Expression of *fdeC* is known to be regulated by environmental conditions in UPEC and Shiga toxin-producing *E. coli* (STEC). The observation in a previous study that an APEC strain IMT5155 in which the *fdeC* gene was disrupted by a transposon insertion resulted in elevated adhesion to chicken intestinal cells prompted us to further explore the role of *fdeC* in infection. We found that the *fdeC* gene prevalence and FdeC variant prevalence differed between APEC and nonpathogenic *E. coli* genomes. Expression of the *fdeC* gene was induced at host body temperature, an infection relevant condition. Disruption of *fdeC* resulted in greater adhesion to CHIC-8E11 cells and increased motility at 42 °C compared to wild type (WT) and higher expression of multiple transporter proteins that increased inorganic ion export. Increased motility may be related to increased inorganic ion export since this resulted in downregulation of YbjN, a protein known to supress motility. Inactivation of *fdeC* in APEC strain IMT5155 resulted in a weaker immune response in chickens compared to WT in experimental infections. Our findings suggest that FdeC is upregulated in the host and contributes to interactions with the host by down-modulating motility during colonization. A thorough understanding of the regulation and function of FdeC could provide novel insights into *E. coli* pathogenesis.

**Supplementary Information:**

The online version contains supplementary material available at 10.1186/s13567-024-01327-5.

## Introduction

Extraintestinal pathogenic *Escherichia coli* (ExPEC) encompasses a wide variety of pathogenic *E. coli* strains that can cause various diseases at non-intestinal sites [1]. These infections range from urinary tract infections (UTIs) to more severe conditions such as sepsis, meningitis, and pneumonia [2, 3]. Avian pathogenic *E. coli* (APEC), a subgroup of ExPEC with zoonotic potential, is a significant pathogen in the poultry industry, causing colibacillosis, which leads to substantial economic losses and affecting animal welfare worldwide [4]. Moreover, ExPEC, including APEC, are becoming increasingly resistant to antibiotics, posing a significant challenge to effective treatment [5, 6].

APEC infections can be viewed as a dynamic, balanced process in which multiple virulence factors play important roles at different stages. During initial colonization, various adhesins are the key determinant of bacterial attachment and invasion [7]. As the infection progresses, toxins and mechanisms of immune evasion play an increasingly important role in bacterial survival and persistence [8]. Nonetheless, expression of virulence factors during infection comes with a cost, and there is an observable trade-off between the benefits of virulence and the fitness costs associated with maintaining these traits [9–11]. However, the interplay between the virulence factors during APEC infection is not yet well understood.

Although adhesins are critical for *E. coli* infection and their deletion usually leads to reduced virulence, numerous studies have shown that disrupted expression of specific adhesins can unexpectedly enhance adhesiveness by upregulating other virulence determinants. For instance, the deletion of the type 1 fimbrial cluster *fim* in uropathogenic *E. coli* (UPEC) strains has been shown to increase adhesion to bladder cells, potentially by increasing the expression of other adhesins, such as the P fimbriae, or by promoting the formation of bacterial clusters [12]. Such observations are not limited to the ExPEC strains. An enterohemorrhagic *E. coli* (EHEC) strain lacking the *fliC* gene exhibited increased adhesion to host cells that may have been due to an observed upregulation of the other fimbriae, although the lack of motility may have also played a role by enhanced binding or retention in the mucus layer [13]. Another study found that the deletion of the major fimbrial subunit gene (*lpfA*) of long polar fimbriae (LPF) in the same pathotype increased adherence to human intestinal explants, further supporting complex interplay between bacterial adhesion systems [14]. This effect is not unique to *E. coli*. In *Salmonella* Typhimurium deletion of the curli fimbriae encoding operons – *csgBAC* or *csgDEFG* led to increased bacterial colonization of lettuce, suggesting compensatory effect of other adhesins or colonization factors [15]. Similar phenomena have also been observed among Gram-positive bacteria, such as *Streptococcus pneumoniae* and *Staphylococcus aureus* [16].

We previously reported that the FdeC autotransporter adhesin plays a significant role in decreasing adherence of *E. coli* to intestinal epithelial cells suggesting an interplay between FdeC and other virulence factor/-s [17]. We therefore investigated the role of FdeC in APEC pathogenicity and determined potential trade-offs associated with its lack of expression. To explore this phenotype further, we conducted comprehensive analyses including bioinformatics, proteomics and molecular biology approaches. We compared the FdeC prevalence and sequence variation among APEC and nonpathogenic *E. coli* genomes. Following this, we utilized adhesion assays of wild-type (WT) and *ΔfdeC* strains to chicken epithelial cells, along with motility assays and proteomic analysis to determine the regulatory factors underlaying the observed changes. Finally, we studied the comparison of host immune responses to a challenge with WT or *ΔfdeC* in a chicken (systemic) infection experiment.

## Materials and methods

### Bacteria, plasmids and growth conditions

All bacterial strains and plasmids used in this work are listed in Table 1 and Table 2, respectively. Unless otherwise indicated, bacteria were routinely cultured for 16 h at 37 °C in dynamic or static conditions in Lysogeny Broth (LB) or LB containing 1.5% agar, respectively. For selection or plasmid maintenance, antibiotics were used at the following concentrations: Ampicillin (Amp) 100 μg/mL; Chloramphenicol (Cm) 25 μg/mL; Kanamycin (Km) 50 μg/mL. For all experiments, overnight (O/N) cultures of bacteria were grown 16 h before the assay. Cell growth was monitored by measuring optical density at 600 nm.

**Table 1:** **Bacterial strains used in this study**

Strain	Relevant feature(s)	Reference
*E. coli* APEC IMT5155 (O2:K1:H5)	wild type	Prof. dr hab. Peter Schierack, BTU Cottbus-Senftenberg, Germany
*E. coli* APEC IMT5155*ΔfdeC*	*E. coli* APEC IMT5155 with *fdeC* gene knockout	This study
*E. coli* MG1655	Nonpathogenic *E. coli* strain *F*-, *lambda*-,* rph-1*	Wroclaw University of Environmental and Life Sciences, Department of Biochemistry and Molecular Biology collection
*E. coli* XL1-Blue	*recA1 endA1 gyrA96 thi-1 hsdR17 supE44 relA1 lac [F´ proAB lacIq Z¨M15 Tn10 (Tetr)]*	Wroclaw University of Environmental and Life Sciences, Department of Biochemistry and Molecular Biology collection
*E. coli* 3078	*E. coli* with nonpathogenic variant of *fdeC* gene	Prof. dr hab. Peter Schierack, BTU Cottbus-Senftenberg, Germany

**Table 2:** **Plasmids used in this study**

Plasmid	Relevant feature(s)	References
pKD46	pBAD λ redαβγ ts ori; AmpR	[18]
pKD4	template plasmids for FRT-flanked kanamycin cassette	[18]
pCP20	helper plasmid FLP ts *ori*; AmpR, KanR	[72]
pBAD33	Expression vector under the arabinose-induced pBAD promoter, CmR	[23]
pBAD33-fdeC-SD	pBAD33 vector with *fdeC* from APEC IMT5155 and Shine Dalgarno sequence, CmR	This study
pBAD33-fdeC-SD-3078	pBAD33 vector with nonpathogenic *fdeC* variant from *E. coli* strain 3078 and Shine Dalgarno sequence, CmR	This study
pFPV25.1GFPmut3.1Kan-2xHA	plasmid with GFPmut3 under the constitutive *rpsM* promoter, multliple cloning site and hemagglutinin tag (HA), KanR	This study
pFPV25.1GFPmut3.1Kan-fdeC-2xHA	pFPV25.1GFPmut3.1Kan-2xHA plasmid with *fdeC* from APEC IMT5155 and promoter sequence	This study

### Cell line and cell culture

The chicken intestinal epithelial cell line CHIC-8E11 was obtained from Karsten Tedin, FU Berlin [17]. Cells were cultured at 37 °C in 5% CO_2_ in Dulbecco’s Modified Eagle Medium/Ham’s F12 (DMEM/Ham’s F12) supplemented 5% bovine serum, L-glutamine, and penicillin–streptomycin and passaged in the log phase of growth (at a confluency of 80–90%) according to standard protocols. For the *E. coli* adhesion assays cells were seeded in 24-wells plate at a density of 1.2 × 10^5^ cells and used in experiments after five, six or seven days.

### Bacterial mutant construction

The knock-out of the *fdeC* gene in *E. coli* IMT5155 was based on the method previously described by Datsenko and Wanner with minor modifications [18, 19]. A kanamycin cassette flanked by FRT sites was amplified by PCR with the use of primers P0044, P0045 (Table 3). Correct allelic replacement and removal of the marker cassette was confirmed by colony PCR using locus-specific primer pairs: P0046, P0047, P0048 (Table 3) and Sanger sequencing (LGC, Germany). To establish whether the isolates showed altered growth rate or morphology compared to the parental isolates, cultures were used to determinate growth curves and were stained with acridine orange to investigate with the use of a fluorescence microscope [20].

**Table 3:** **Primers used in this study**

Name	Sequence (5′-3′)	References
P0044	ATG TCA CGT TAT AAA ACA GAC AAT AAA CAG CCA CGA TTT CGT TAT TCA GTG TGT AGG CTG GAG CTG CTT C	[18]; This study
P0045	TTA TTT CTC CTC AGC GCC TTC AGT ATC TGC AGG AAC GGC GTT AAT TGT CAC ATA TGA ATA TCC TCC TTA G	[18]; This study
P0046	AGG AAA CCG GGA AAT CCC C	This study; CP005930.1 3251820- 3256070
P0047	TTC GCC CGG TTT AGC CTG	This study; CP005930.1 3251820- 3256070
P0048	CGA AGA GTC CTT CAG CGA G	This study; CP005930.1 3251820- 3256070
O1646	CAG TCA TAG CCG AAT AGC CT	[18]
O1647	CGG TGC CCT GAA TGA ACT GC	[18]
P0152	ACA TGG TAC CAG GAG GAC AGC TAT GTC ACG TTA TAA AAC AGA CAA T	This study; CP005930.1 3251820- 3256070
P0116	ACA TTC TAG ATT ATT TCT CCT CAG CGC CTT C	This study; CP005930.1 3251820- 3256070
P0169	ACA TGG TAC CAG GAG GAC AGC TAT GTC GCG TTA TAA AAC AGG TC	This study; CP005930.1 3251820- 3256070
P0170	ACA TTC TAG ATT ATT TCA TCG CCT CCT CGT C	This study; GCA_018431385.1 JAGUDJ010000013.1:81090–81111
MCS_2xHAfor	CCC CGG GAG ATC TGT CGA CTA CCC ATA CGA TGT TCC AGA TTA CGC TTA CCC ATA CGA TGT TCC AGA TTA CGC TTA AG	This study; [73]
MCS-2xHArev	GAT CCT TAA GCG TAA TCT GGA ACA TCG TAT GGG TAA GCG TAA TCT GGA ACA TCG TAT GGG TAG TCG ACA GAT CTC CCG GGG AGC T	This study; [73]
P0184	ACA GAG CTC AAG TAT CTA TTT AAT GAC TTG CAC AAA AAG	This study; CP005930.1 3251820- 3256070
P0185	ACA GAT CTT TTC TCC TCA GCG CCT TCA G	This study; CP005930.1 3251820- 3256070

Underlined sequences represent restriction sites. Accession numbers corresponding to gene templates can be found in the references.

### Cloning

To construct a vector capable of expressing the FdeC-hemagglutinin fusion protein to monitor *fdeC* promoter activity by measuring GFP expression at the same time, sequences of 2xHA and multiple cloning site (SacI, SmaI(XmaI), BglII, SalI) was cloned into the pFPV25.1Kan plasmid. Primers MCS_2xHAfor and MCS-2xHArev (Table 3) were PAGE purified and hybridized. Briefly, oligos were resuspended in hybridization buffer (10 mM Tris, pH 7.5–8.0, 50 mM NaCl, 1 mM EDTA) and processed in thermocycler at four steps: (1) 95 °C—2 min; 2) 95 °C—20 s; (3) back to step 2 − 0.5 °C /repeated 39 times/ final temp.- 25 °C; (4) 4 °C. Hybridized oligos were next cloned into the pFPV25.1Kan plasmid in the SacI/BamHI digestion sites. The APEC and nonpathogenic variant of the *fdeC* gene was amplified from *E. coli* IMT5155 and *E. coli* 3078, respectively [21, 22]. Amplification was carried out by PCR using *Phusion* polymerase (Thermo) with following primer pairs P0152 and P0116, P0169 and P0170, P0184 and P0185 (Table 3). PCR products were purified by the GeneJET PCR Purification Kit (Thermo) whereas plasmid DNA was isolated by the GeneJET Plasmid Miniprep Kit (Thermo). For cloning, the *fdeC* gene was inserted into the pBAD33 plasmid in KpnI/XbaI digestion sites, while for the pFPV25.1GFPmut3.1Kan-2xHA *fdeC* with promoter sequence was cloned into SacI/BglII sites. DNA sequence of all the inserts was confirmed by Sanger sequencing (LGC, Germany).

### Adhesion assays

For infection of cultures cells, IMT5155 WT and IMT5155Δ*fdeC* were cultured O/N in 1 mL LB medium at 37 °C with shaking (180 rpm). Cultures were diluted to OD_600_ = 0.05 and grown at the same temperature with shaking (220 rpm) to early stationary growth phase (OD_600_ = 2.0). For assays performed with APEC IMT5155, IMT5155Δ*fdeC,* IMT5155Δ*fdeC* + pBAD33-*fdeC*-IMT5155, IMT5155Δ*fdeC* + pBAD33-*fdeC*-3078 and IMT5155Δ*fdeC* + pBAD33, bacteria were grown O/N in 1 mL LB pH 8.0 medium at 42 °C (with Cm for pBAD33 plasmid selection), 180 rpm. Next day, cultures were diluted to OD_600_ = 0.05 in LB pH 8.0 (with Cm for pBAD33 plasmid selection) and grown to mid-log growth phase (OD_600_ = 0.5) at 42 °C, 220 rpm. Next, P_BAD_ promoter expression was induced for 3 h by addition of 0.2% L-arabinose at the same conditions [23]. Bacteria were washed and diluted to a concentration of 1.16 × 10^7^/mL (MOI = 100) and were incubated with the CHIC-8E11 cells for 2 h at 37 °C (for bacteria grown at 37 °C) or 42 °C (for bacteria grown at 42 °C) in 5% CO_2_. When indicated, a centrifugation step (10 min, 1000 × *g*) was carried out to bring bacterial and epithelial cells into close contact and thereby to enable bacteria to initiate infection [24]. After the 2-h incubation step, the medium was removed, and cells were washed three times with PBS and lysed with 1% Triton X-100 in PBS. The number of CFU in each well was determined by plating serial dilutions of cell lysates on LB agar. At least three independent experiments with three technical replicates for each *E. coli* strain were performed.

### Reporter gene assays

APEC IMT5155 WT was transformed with pFPV25.1GFPmut3.1Kan-fdeC-2xHA construct by electroporation according to the Sambrook and Russell protocol [25]. For assays, transformants were grown O/N at 37 °C, 180 rpm. Cultures were diluted to an OD_600_ = 0.05 and grown in alkaline (pH = 8.0) or acidic (pH = 5.8) LB at 37 °C or 42 °C. Once the cultures reached early exponential (OD_600_ = 0.5), late exponential (OD_600_ = 1.0), and early stationary (OD_600_ = 2.0) growth phase, the equivalent of bacteria at OD_600_ = 0.4 was taken in each of measuring points and centrifuged for 4 min at 4 °C, 16 100 × *g* [26]. For Western Blot analysis, pellets were suspended in 100 µL of loading dye (50 mM Tris–HCl pH 6.8, 2% SDS, 10% glycerol, 1% β-mercaptoethanol, 12.5 mM EDTA, 0.02% bromophenol blue), incubated 5 min at 95 °C and subjected to SDS PAGE in a 10% gel (consisting of 4% stacking and 10% resolving gel composed of Tris–HCl pH 6.8 and 8.8, respectively, acrylamide:bisacrylamide, SDS, APS and TEMED). The separated proteins were transferred onto a nitrocellulose membrane and blocked 1 h at RT with 5% fat-free dry milk in PBST (PBS supplemented with 0.1% Tween-20). HA-Tag Rabbit mAb (Cell Signalling, C29F4) was used as a primary antibody in 1:1000 dilution, whereas the secondary antibody Anti-rabbit peroxidase (Sigma, A6154) was used in 1:5000 dilution. The blots were developed with Clarity Western ECL Substrate (BioRad). After the first blotting, the membrane was incubated with 30% H_2_O_2_ for 20 min at 37 °C to inactivate peroxidase activity [27]. Then, the membrane was washed two times with PBST and processed with Western Blot as described above, however with GFP Mouse mAb in 1:1000 dilution as a primary antibody (Cell Signalling, 4B10) and a secondary antibody Anti-mouse peroxidase (Dako, P0447) was used in 1:5000 dilution. The Western Blots were developed with the use of Chemidoc XRS + and analyzed using Image Lab software (BioRad).

### Motility assays

APEC IMT5155 WT, IMT5155Δ*fdeC*, IMT5155Δ*fdeC* + pBAD33-*fdeC*-IMT5155, and IMT5155Δ*fdeC* + pBAD33 were grown O/N in 1 mL of LB (with Cm for pBAD33 plasmid selection). The optical density was measured, bacteria were diluted to OD_600_ = 1 and 1.5 μL of each strain was inoculated into the centre of plates with LB (with 1% NaCl) or low-salt LB (with 0.05% NaCl) containing 0.25% agar [28]. For transformants with pBAD33 plasmid, Cm and 0.2% L-arabinose was added to induce the P_BAD_ promoter. All plates were incubated at 37 °C or 42 °C for 16 h. The diameter of the diffusion zone from the point of inoculation was measured with a ruler. At least three independent experiments with three repetitions for each *E. coli* strain were performed.

### Sample preparation for proteomic analysis

The APEC IMT5155 WT and IMT5155Δ*fdeC* strains were cultured O/N in LB pH 8.0, 180 rpm at two different temperatures: 1) 37 °C (non-inducing conditions for *fdeC* expression) or 2) 42 °C (inducing conditions for *fdeC* expression). Next, the cultures were diluted in the same medium to an OD_600_ = 0.05. The cultures were then grown under similar conditions, with shaking at 220 rpm, until reaching an OD_600_ = 0.5. Afterwards, the cultures were grown for an additional 3 h at the same conditions. The bacteria were cooled on ice, pelleted by centrifugation at 4 °C, and washed twice with ice-cold PBS. The resulting pellets were immediately frozen at −80 °C and prepared for proteomic analysis as described previously by Schmidt et al. [29] with minor modifications. Briefly, bacterial cell pellet was lysed with 2% sodium deoxycholate, ultrasonicated and heated to 95 °C. The extracted proteins were measured with the use of a BCA protein assay kit (Thermo). Next, proteins were reduced with tris(2-carboxyethyl)phosphine (TCEP), alkylated with chloroacetamide and digested with LysC and trypsin, according to Glatter et al. [30]. The peptide mixture was desalted with trifluoroacetic acid (TFA), followed by drying with a centrifugal evaporator. The dried peptides were resuspended in 0.1% formic acid to a concentration of 0.4 μg/μL. In each LC–MS/MS measurement 2 µg of total peptide amount was injected onto the nano-HPLC system.

### DIA mass spectrometry and DIA data analysis

LC–MS/MS runs in DIA mode were performed on an Orbitrap Exploris-480 (Thermo Scientific). Peptides were separated using a 90 min gradient from 2 to 42.5% buffer (0.1% formic acid, 90% acetonitrile). A 30-variable window DIA scheme was applied, covering the precursor mass range of 360–1370 m/z with a total cycle time of ~4 s. Total run time was 110 min.

Data were analyzed with the DIA-NN [31] software against an *E. coli* APEC IMT5155 (Uniprot: UP000031137) fasta database. Minimal peptide length was set from 7–30 amino acids, with a single missed cleavage allowed. Carbamidometthylation of cysteines was set as fixed modification while oxidation of methionine was considered as variable modification. Peptides and proteins were filtered at 1% false discovery rate (FDR). Differential analysis was performed with Limma test, using a log_2_ fold change cut off > 1. Differentially expressed proteins were functionally annotated using eggNOG-mapper ver. 2.13 and assigned functions/categories according to the Cluster of Orthologous Genes (COGs) database [32]. Statistical analysis was carried out using the R statistical software. The frequency of statistically significant genes in proteomics analysis within COG categories was compared between strains and conditions with the Chi-squared test of independence implemented in R [33]. Additionally, the GSEA function from clusterProfiler package was used to perform a gene set enrichment analysis for COG categories [34]. COG categories were considered significantly upregulated/downregulated if the Benjamini–Hochberg corrected *p* value was equal to or lower than 0.05. The mass spectrometry proteomics data have been deposited to the ProteomeXchange Consortium via the PRIDE [35] partner repository with the dataset identifier PXD045075 (Username: reviewer_pxd045075@ebi.ac.uk, Password: 0cH3ZcmY).

### FdeC sequence variation determination

APEC genomes were searched in BioProjects registered at NCBI (573) and PubMed database (10, single “reference” APEC genomes). The full list of BioProjects and single genomes are available in Additional file 1. Genomes of APEC were deposited in GenBank as assemblies or raw reads. Where possible assembled genomes were downloaded. Sequencing reads available in fastq format for the rest of the genomes were downloaded, assembled with Shovill and annotated with Prokka [20, 36]. All assemblies with more than 600 contigs were removed from analysis. A set of 3069 nonpathogenic *E. coli* genomes was downloaded and analysed as described by Kolenda et al. [22]. Sequences of the *fdeC* gene were found and downloaded from APEC and nonpathogenic *E. coli* genomes with BLAST [37]. Obtained sequences were translated with the Ugene software [38]. Next, primary structures of FdeC variants were clustered with the use of CD-HIT with settings that proteins considered as one variant/cluster had to have 100% sequence identity and coverage [39]. All variants found in less than 10 APEC or nonpathogenic *E. coli* genomes were re-clustered as the group “Other”. Genomes with no *fdeC* sequence were re-clustered as the group “Not present”. FdeC-based phylogeny was constructed with the use of RAxML version 8.2.12 with 43 most frequent FdeC variant sequences, GAMMJTT substitution model and 500 bootstraps. The tree was annotated with the use of iTOL [40].

### Animal infection study

APEC IMT5155 WT and IMT5155Δ*fdeC* strains were cultured in FdeC-inducing conditions as for the in vitro adhesion assays. Briefly, overnight cultures were grown in LB pH = 8.0 at 42 °C, 180 rpm. Next day, cultures were diluted to OD_600_ = 0.05 in LB pH 8.0 and grown to mid log growth phase (OD_600_ = 0.5) at 42 °C, 220 rpm. Next, bacteria were grown for an additional 3 h at the same conditions. Then, bacteria were washed and resuspended in PBS. OD_600_ was measured and bacteria were diluted to concentrations of 10^9^ per 0.5 mL of PBS (single dose for one animal). As a control, serial dilution was prepared, and bacteria were spread on LB agar to confirm the CFU. Five-week-old SPF White Leghorn chickens line AU L22 B^15^ were reared in the animal facilities at Aarhus University with ad libitum access to the feed and water. Chickens were free of distinct pathogens and kept in a biosecure facility. The virulence of APEC IMT5155 was tested in a systemic chicken infection model, as described by Antão et al. [21]. Briefly, 75 chickens were inoculated intratracheally with 0.5 mL of PBS suspension containing 10^9^ CFU of IMT5155 WT (30), IMT5155Δ*fdeC* (30) or no *E. coli* (15). The chickens inoculated with only PBS were used as negative controls. At six, 24, and 48 h post-infection, chickens were euthanized and bacterial loads were investigated in lungs, livers and spleens. The organs were homogenized in sterile PBS with one sterile stainless 5 mm metal bead and TissueLyser II (Qiagen) and the homogenates were diluted and plated onto LB agar for CFU determination [41]. Tissue samples (lungs and spleens) for RT-qPCR were transferred to RNAlater and stored at −80 °C. Additionally, at 24 and 48 h post-infection, immediately before euthanasia, blood samples were collected and proceed with flow cytometry as described below. At 48 h post-infection whole chickens and their left lung was weight after euthanasia.

### Flow cytometry

Enumeration of different cell subsets in peripheral blood were determined using a no-lyse no-wash flow cytometric method as previously described by Seliger et al. [42] with a few modifications. Fifty microliters of heparin stabilized blood (pre-diluted 100 times with Fluorescence-activated cell sorting (FACS) buffer (0.2% BSA, 0.2% sodium azide, 0.05% normal horse serum in PBS) was mixed with 50 μL of antibody master mix (Panel 1 or Panel 2 in Additional file 2) in 96-well U-bottom plates. Staining was done for 20 min in darkness at 4 °C and immediately before acquisition eBeads™ CountingBeads (Thermofisher, cat. No. 01–1234-42), diluted 1:10 in FACS buffer and 4 mM EDTA were added. Forty microliters of each sample were acquired in the flow cytometer. Absolute number of cells was calculated according to the eBeads™ manufactures instructions. Staining panel 1 identified: monocytes, thrombocytes and heterophils. Staining panel 2 identified: B cells, CD8 + γδ T cells, CD8- γδ T cells, CD4 + αβ T cells, CD8 + αβ T cells and CD4 + CD8 + αβ T cells (double positives). All flow cytometric analyses were conducted on a BD FACSCelesta™ flow cytometer with a flow rate of 0.5 μL per second and analyses of acquired samples were performed in the FACS Diva software.

### RNA extraction and cDNA synthesis

Total RNA was isolated from collected lungs and spleens stored in RNAlater. To each 250 mg of tissue, 600 µL of RLT buffer with β-ME was added and the tissues were disrupted using a Tissue Lyser LT (2 × 3 min, 30 Hz). Total RNA was isolated using a RNeasy Mini Kit (Qiagen) according to manufacturer’s protocol. The purity and amount of extracted total RNA were assessed using a NanoDrop™ 1000 device. The 250 ng of total RNA was used to generate cDNA from the collected tissues. First-strand cDNA synthesis was carried out using a SuperScript III reverse transcription kit (Thermo) including random primers according to manufacturer’s instruction.

### Determination of immune gene expression by multiplexed qPCR

Highly multiplexed qPCR analysis of an immune gene panel consisting of 89 genes and 7 reference genes for data normalization was conducted as described by Borowska et al. [43]. To increase the template cDNA yield, a preamplification reaction was performed prior to the microfluidic qPCR. Preamplification was conducted with the use of TaqMan PreAmp Master Mix. A mixture of 200 nM primers was prepared by mixing an equal concentration of all the primers used in the multiplex PCR. For the PCR, 10 μL of TaqMan PreAmp Master Mix was mixed with 5 μL of the 200 nM primer mixture and 5 μL of diluted cDNA (at a concentration of 185 ng/μL) in a 1:7 ratio. The samples were then incubated at 95 °C for 10 min, followed by 14 cycles of 95 °C for 15 s and 60 °C for 4 min. To remove any unincorporated primers from the preamplified cDNA, a clean-up step was performed using Exonuclease I (*E. coli*) (New England Biolabs) according to the instructions from the manufacturer. qPCR was conducted using a BioMark HD instrument and the 96.96 Dynamic Array (Fluidigm). For the assay mixes, 2.5 μL of 2X Assay Loading Reagent (Fluidigm), 2.3 μL of a primer pair mixture (with a final concentration of 1.15 μM), and 0.2 μL of low EDTA TE buffer were combined. For the sample mixes, 2.5 μL of TaqMan Gene Expression Master Mix (Applied Biosystems), 0.25 μL of 20X DNA Binding Dye Sample Loading Reagent (Fluidigm), 0.25 μL of 20X EvaGreen DNA binding dye (Biotum), and 2 μL of the preamplified cDNA were mixed. The qPCR was performed under the following conditions: initial thermal mix at 50 °C for 2 min, 70 °C for 30 min, 25 °C for 10 min, followed by a hot start at 50 °C for 2 min and 95 °C for 10 min. The qPCR ran for 30 cycles at 95 °C for 15 s, 60 °C for 60 s, terminated with a melting curve analysis from 60 °C for 3 s up to 95 °C. The following parameters were used in the analysis: the quality threshold was adjusted to 0.65, the baseline correction to linear and the quantification cycle (Cq) threshold method was set to automatic.

### Fluidigm data analysis

qPCR data pre-processing, normalisation, relative quantification and statistics were performed in GenEx5 and GenEx Enterprise (MultiD Analyses AB). Data were corrected for reaction efficiency for each primer assay individually. The most stably expressed reference gene for the combined tissue dataset were identified from a panel of seven reference genes to be GUSB and this was used to normalise all samples in GenEx5. Relative quantities were transformed to logarithmic scale (log_2_) before statistical analysis, which was performed with the use of a t-test. The principal component analysis (PCA) was run in R with RStudio. Gene expression differences were considered significant (*p* value ≤ 0.05) for fold change values lower than -1 and higher than 1.

### Figures and statistical analysis

The ggplot2 package implemented in R software was used to generate the figures [33, 44]. Adhesion and motility levels of different *E. coli* isolates was compared with t test implemented in R package. FdeC variant prevalence was compared with Chi squared test of independence implemented in R package [45].

## Results

### Deletion of *fdeC* increases adhesion of APEC IMT5155 to intestinal epithelial cells in infection-relevant conditions

**Figure 1:**
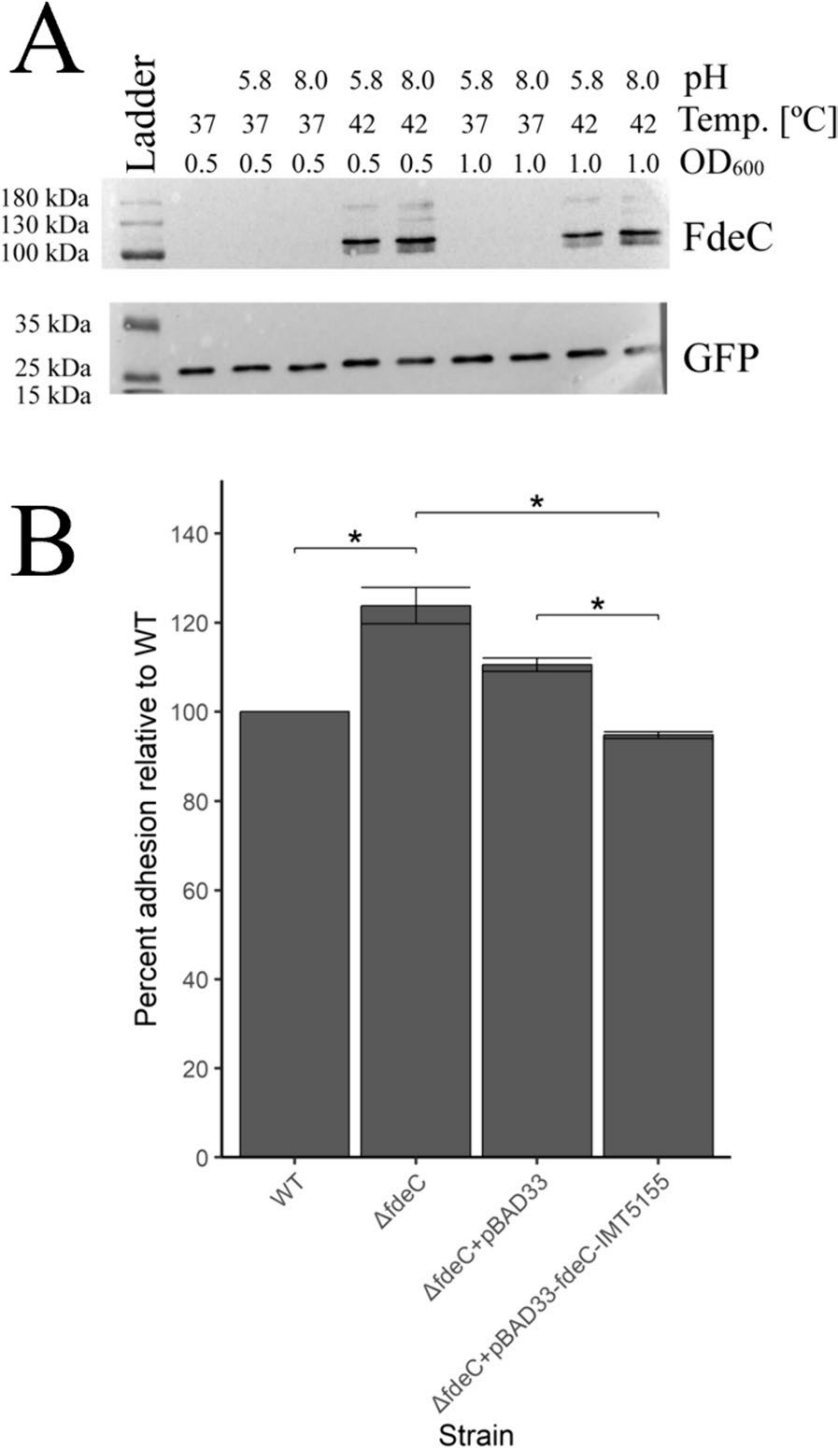
**Role of FdeC in adhesion of APEC IMT5155 to chicken intestinal epithelial cells.** **A** Determination of FdeC expression inducing conditions by Western Blotting. Protein molecular weight marker (kDa), OD_600_- phase of growth (0.5, 1.0), Temperature- 37 °C or 42 °C, pH-, not adjusted (empty space), 5.8 or 8.0; Full size Western blotting images are presented in Additional file 3) Adhesion assay performed in FdeC expression inducing conditions. Names of isolates used for adhesion to CHIC-8E11 cells are shown on the x-axis. The percent of isolate’s adhesion relative to IMT5155 WT is shown on y-axis. Error bars show median absolute deviations from at least three experiments. All statistically significant comparisons are shown. Statistically significant observations with *p* value < 0.05 are marked with “*”.

To assess the role of FdeC in adhesion of APEC, initially IMT5155 wild type (WT) and *fdeC* deletion mutant (Δ*fdeC*) were incubated with chicken intestinal epithelial cells CHIC-8E11 at 37 °C. Consistent with previous reports that expression of FdeC is induced at higher temperature and alkaline pH in EHEC [46], we did not observe any significant differences in adhesion between these two strains at 37 °C (data not shown). We confirmed a similar regulation of *fdeC* in APEC IMT5155 using a hemagglutinin Epitope Tag-based (HA-Tag) reporter system (Figure 1A, Additional file 3). Next, we performed the adhesion assay with bacteria grown in FdeC-inducing conditions and observed that adhesion of Δ*fdeC* was higher than WT (Figure 1B, *p* < 0.05). Complementation of the *fdeC* gene *in trans* resulted in return of the adhesive phenotype to the wild-type strain levels (compare Δ*fdeC* with Δ*fdeC* + pBAD33-*fdeC*-IMT5155 and Δ*fdeC* + pBAD33 vs Δ*fdeC* + pBAD33-*fdeC*-IMT5155, Figure 1B, *p* < 0.05). No statistically significant differences in adhesion were observed when WT was compared with Δ*fdeC* + pBAD33-*fdeC*-IMT5155.

### Deletion of *fdeC* increases motility of APEC IMT5155

**Figure 2:**
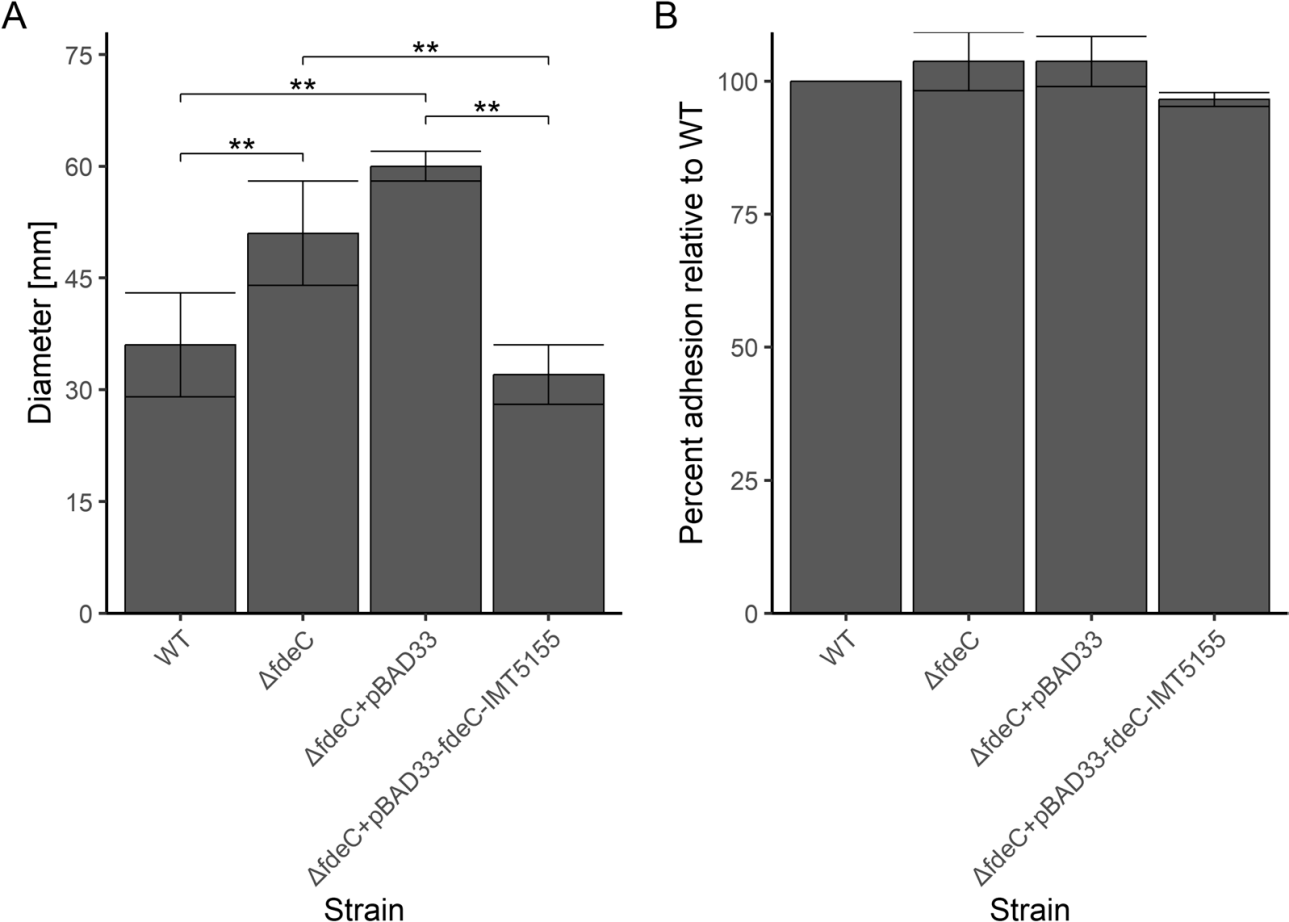
**Influence of FdeC expression on APEC IMT5155 motility.** **A** Motility assays performed in FdeC expression inducing conditions. Names of isolates used for assay on semi solid agar are shown on the x-axis. The median diameter of growth is shown on the y-axis. Error bars show median absolute deviations from at least three experiments. All statistically significant comparisons are shown. Statistically significant observations with *p* value < 0.001 are marked with “**”. **B** Adhesion assay performed in FdeC expression inducing conditions and involved plate centrifugation. Names of isolates used for adhesion to CHIC-8E11 cells are shown on the x-axis. The percent of isolate’s adhesion relative to IMT5155 WT is shown on y-axis. Error bars show median absolute deviations from at least three experiments.

As bacterial adhesion and motility are co-regulated processes [47], we investigated how FdeC expression influences IMT5155 motility. IMT5155Δ*fdeC* and IMT5155Δ*fdeC* + pBAD33 had increased motility compared to IMT5155 (*p* < 0.001) and expression of FdeC from pBAD33 restored the phenotype of the WT strain level at 42 °C (Figure 2A). No differences in motility were observed at 37 °C (data not shown). Next, we performed adhesion assays with centrifugation of bacteria to ensure contact with CHIC-8E11 cells directly after adding bacteria to the cell monolayer and we observed that there were no significant differences in adhesion levels between analysed isolates (Figure 2B). Taken together, our results indicate that FdeC expression reduces bacterial motility, which might be a reason for the impaired ability of bacteria to reach the host cells in our in vitro adhesion model.

### FdeC affects expression of inorganic ion transporters and regulation of motility through NaCl broth concentration

**Figure 3:**
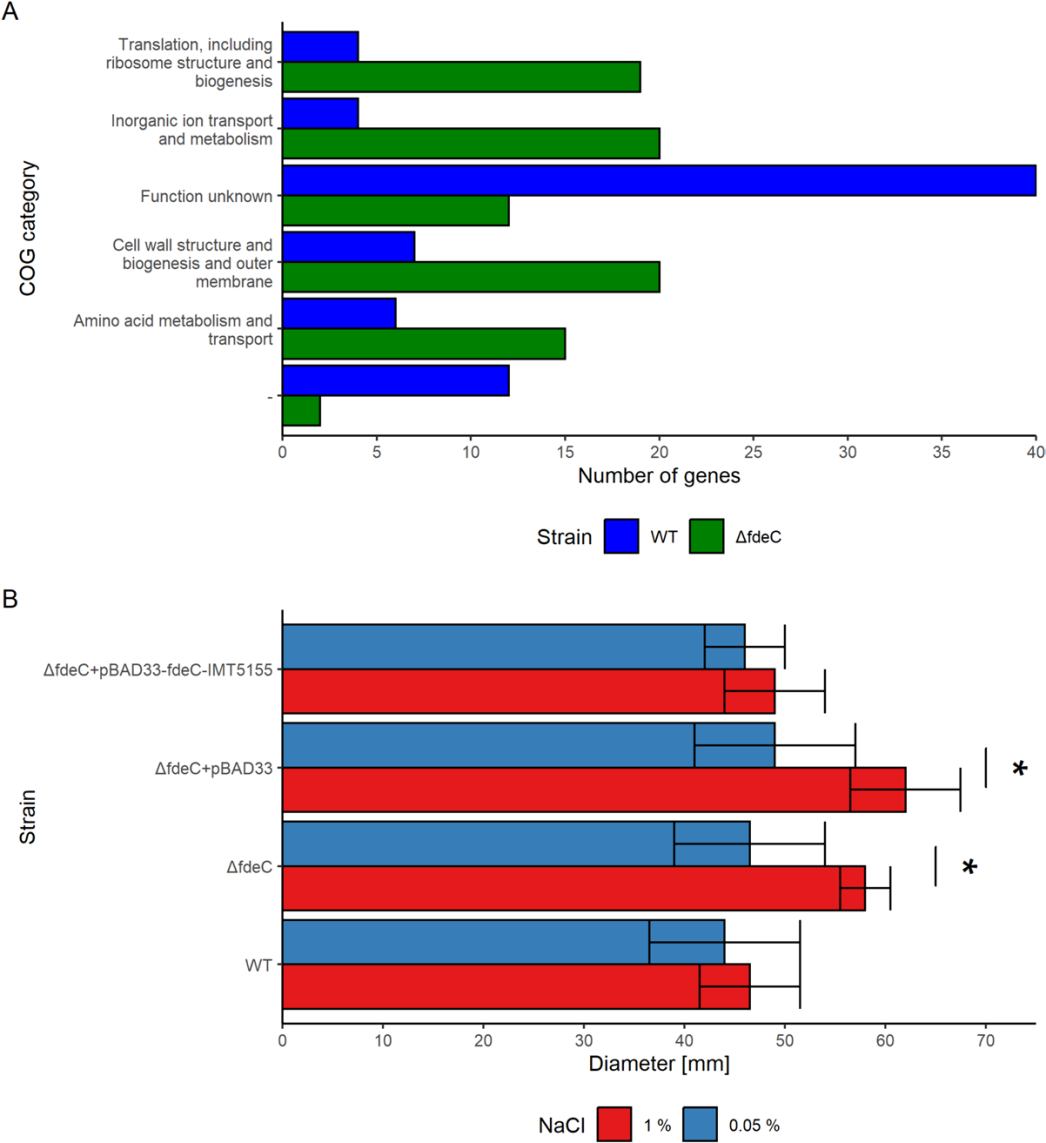
**The role of sodium chloride concentration on motility of IMT5155.** **A** Differentially expressed proteins grouped in Clusters of Orthologous Groups (COGs). COGs were compared between WT and Δ*fdeC* strains grown at 42 °C. The x-axis shows the number of proteins with higher expression in Δ*fdeC* (green bars) or WT (blue bars) strain, and the y-axis shows the COG categories that differed significantly in protein counts between the WT and Δ*fdeC* strains. NA category (“−”) corresponds to proteins that were not assigned to any of the COGs. **B** Motility assays performed on LB agar containing different concentrations of sodium chloride. The median diameter of growth is shown on the x-axis, and the names of isolates used for the assay on semisolid agar are shown on the y-axis. Error bars show median absolute deviations from at least three experiments. Statistically significant observations with *p* value < 0.05 are marked with “*”.

To investigate the underlying molecular mechanisms leading to increased motility after *fdeC* deletion, the proteome of WT and Δ*fdeC* strains grown at 37 °C or 42 °C were analysed by LC–MS/MS. Comparison between the Δ*fdeC* strain and WT at 42 °C revealed that 172 and 150 proteins were up- and downregulated, respectively (Additional file 4, FDR < 0.05). These proteins belonged to various COG categories, but six groups contained significantly different number of proteins with higher expression in Δ*fdeC* or WT strain at 42 °C (*p* < 0.05, Figure 3A). Gene groups upregulated in Δ*fdeC* encoded proteins involved with inorganic ion transport and metabolism, translation, cell wall structure and outer membrane or amino acid metabolism and transport. The majority of proteins upregulated in WT had unknown function. Furthermore, determination of gene set enrichment indicated that proteins responsible for inorganic ion transport and metabolism were upregulated in the Δ*fdeC* strain (Additional file 5). Among proteins upregulated in the Δ*fdeC* strain there was NhaB (Additional file 5), which is responsible for transport of sodium ions across the membrane [48]. It has been previously shown that NaCl concentrations in the media can affect *E. coli* motility [49], hence, we evaluated whether reducing the NaCl concentration in LB semi-solid agar from 1% to 0.05% influenced the motility of the *ΔfdeC* strain. Our findings suggest that a reduced NaCl concentration of 0.05% in the agar indeed diminishes the movement of the *ΔfdeC* strain (*p* < 0.05, Figure 3B).

### FdeC sequence variants encoded in avian pathogenic and nonpathogenic *E. coli* differ in adhesion to epithelial cells

**Figure 4:**
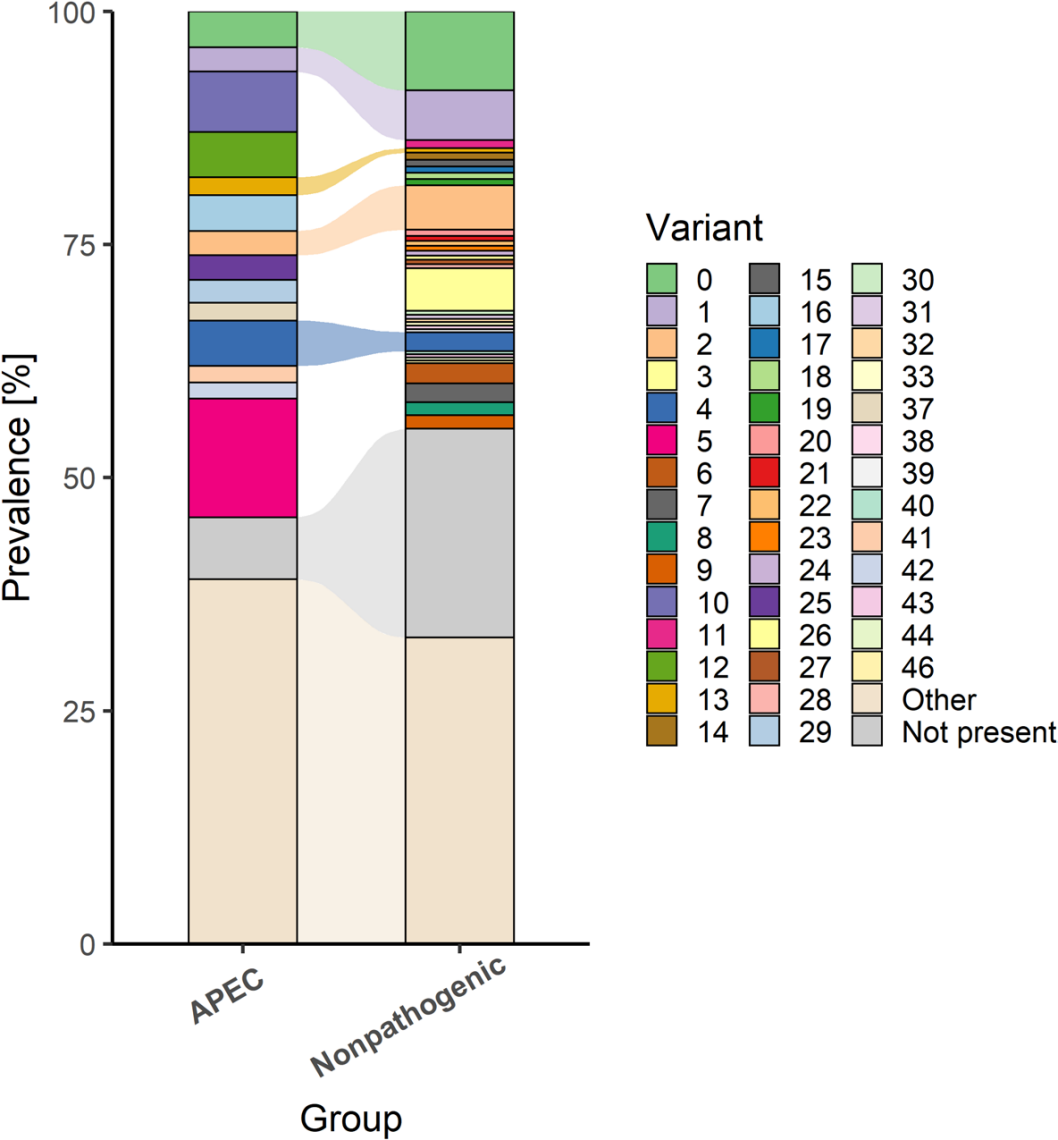
**Prevalence of FdeC variants in APEC and nonpathogenic *E. coli*** Alluvial plot with analysis of FdeC sequence in 573 APEC and 3069 nonpathogenic *E. coli* (a set of *E. coli* genomes that did not possess VAFs typical for well-defined *E. coli* pathotypes). All variants found in less than 10 APEC or nonpathogenic *E. coli* genomes were re-clustered as group “Other”. Genomes with no *fdeC* sequence were re-clustered as group “Not present”. Names of groups used for analysis are shown on the x-axis. The prevalence of each FdeC variant is shown in percent on the y-axis.

**Figure 5:**
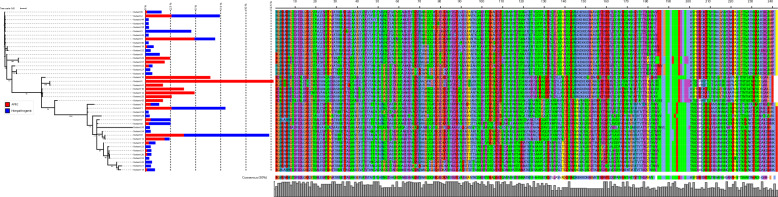
**Sequence analysis of FdeC variants.** Phylogenetic tree based on all FdeC variants present in 10 or more isolates of APEC or nonpathogenic *E. coli*. Prevalence in APEC (red) and nonpathogenic *E. coli* (blue), and FdeC variable sites were annotated as rectangles on the tree with the use of iTOL. One variant was not included in the analysis due to its short sequence (it was a truncated FdeC variant present in 22 isolates of nonpathogenic *E. coli*).

It has been shown for some adhesins that sequence variation can alter the adhesive properties of these proteins [50]. Therefore, we investigated FdeC sequence variation among APEC and nonpathogenic *E. coli*. There were 125 and 630 FdeC variants in 573 APEC and 3069 nonpathogenic *E. coli* genomes, respectively. Importantly, only 15 APEC FdeC variants and 35 nonpathogenic *E. coli* FdeC variants were present in 10 or more genomes (Figure 4). Among these, nine variants were identified in 220 APEC isolates (38.4%) but were absent in nonpathogenic *E. coli*. Conversely, 19 variants were found in 427 nonpathogenic *E. coli* genomes (13.9%) and were not present in APEC (Figure 5). Overall, 83 variants were exclusive to 363 APEC isolates (63.4%), and 588 variants were unique to 1364 nonpathogenic *E. coli* genomes (44.4%). Phylogenetic analysis of the most prevalent FdeC variants revealed that variants unique to APEC clustered together and differed in their variable sites (Figure 5, Additional file 6). Adhesion assays revealed that variant 6 from nonpathogenic *E. coli* (Δ*fdeC* + pBAD33-*fdeC*-3078) showed lower adhesion compared to variant 25 from APEC IMT5155 (Δ*fdeC* + pBAD33-*fdeC*-IMT5155) (Additional file 7, *p* < 0.05).

### Infection of chickens with IMT5155 WT and Δ*fdeC* strain elicits different immune response profiles

**Figure 6:**
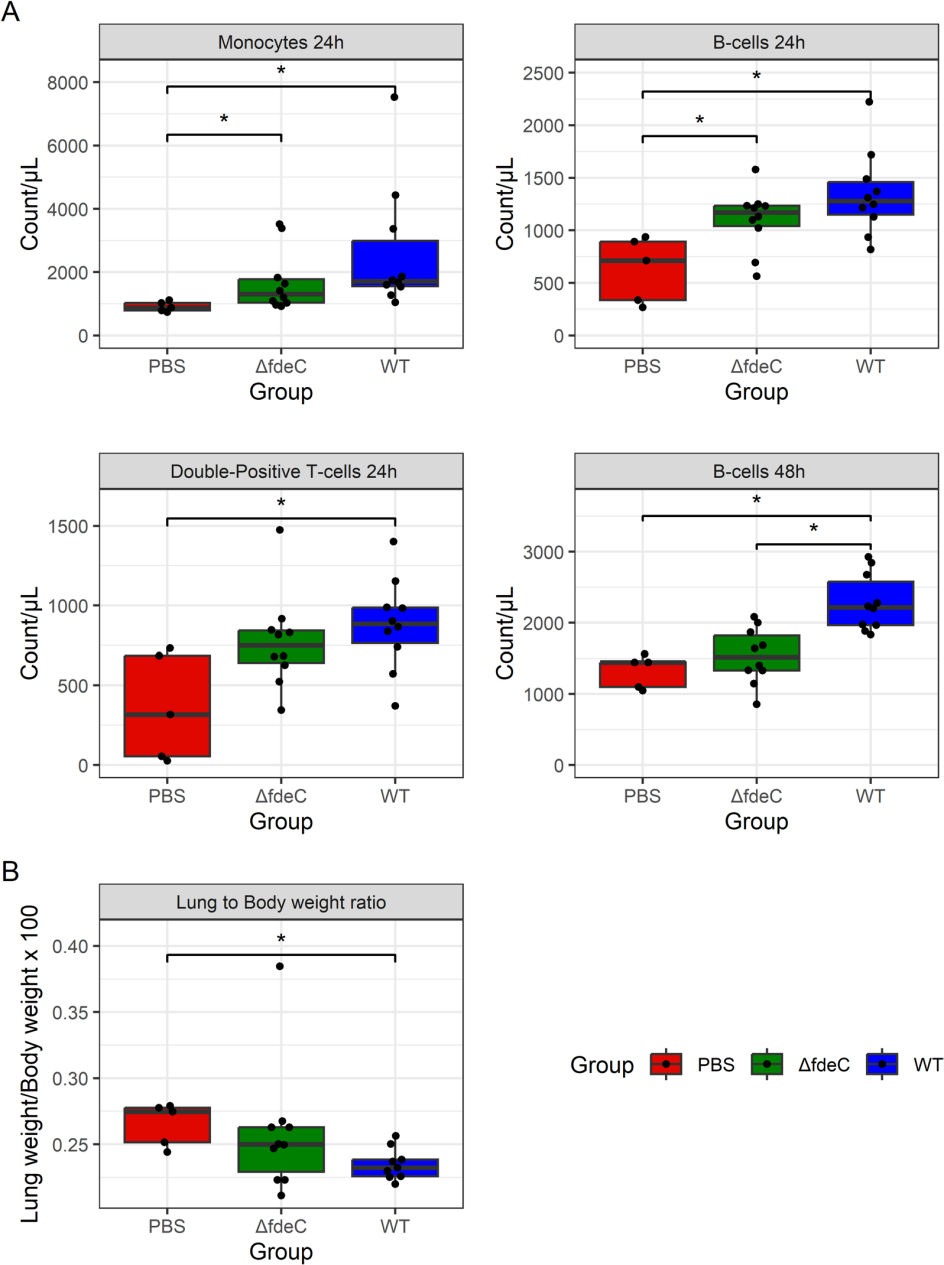
**Changes in white blood cells during chicken infections with APEC strains.** **A** Dot boxplots with results for peripheral blood flow cytometry of chickens 24 or 48 h after intratracheal inoculation with WT, Δ*fdeC* strains or PBS. Counts of cells per microliter peripheral blood are shown on the y-axis and inoculation groups on the x-axis. **B** Dot boxplots with results for left lung to whole body weight ratio of chickens 48 h after intratracheal inoculation with WT, Δ*fdeC* strains or PBS. Left lung to whole body weight ratio (multiplied 100 ×) are shown on the y-axis and inoculation groups on the x-axis. All statistically significant comparisons are shown. Statistically significant differences between groups are marked with “*” (*p* < 0.05, Benjamini Hochberg corrected Wilcoxon test).

The ability of WT and Δ*fdeC* to colonize chickens was investigated by utilizing a chicken systemic infection model. For the majority of the chickens, colonization by the APEC strains was below the detection limit of the CFU determination method employed in this study (Additional file 8). However, peripheral blood white blood cell populations from chickens inoculated with the WT, Δ*fdeC* strains or PBS differed significantly (Figure 6A). Increased counts of monocytes and B-cells following infection with APEC was observed compared to PBS controls after 24 h (*p* < 0.05). Moreover, double positive T cells (CD4 + CD8α +) increased in the blood of chickens after infection with WT strain when compared with PBS controls (*p* < 0.05). Higher counts of B-cells were observed in chickens infected with the WT strains compared to chickens infected with the Δ*fdeC* strain or the negative controls (*p* < 0.05) after 48 h. To test the influence of APEC strains on lung weight, the left lung to body weight ratio was investigated (Figure 6B), which revealed a lower weight of the left lung in chickens treated with WT APEC in comparison to PBS treatment after 48 h (*p* < 0.05). No differences in left lung weight were found between the Δ*fdeC* and PBS treatment.

**Figure 7:**
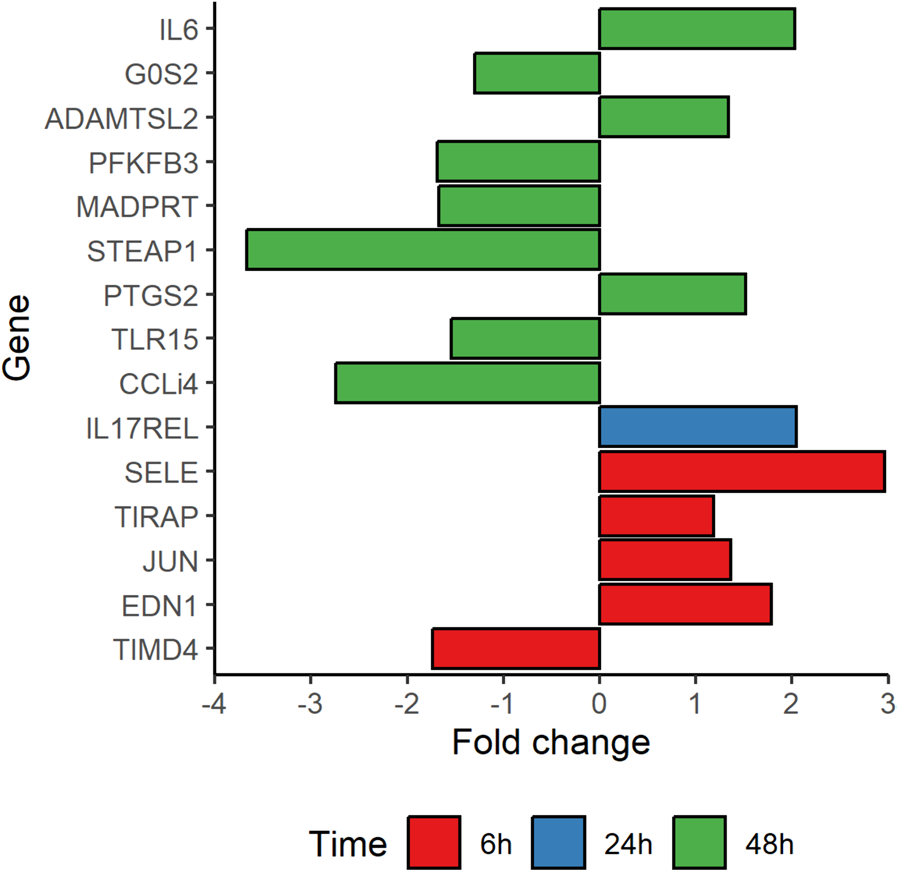
**DEGs observed in chickens infected with APEC strains.** Barplot with fold change difference of genes significantly differentially expressed (*p* < 0.05) in lungs of chickens 6 h (red bars), 24 h (blue bars), 48 h (green bars) after intratracheal inoculation with WT or Δ*fdeC*. Fold change of comparison WT vs Δ*fdeC* are shown on the x-axis. Gene name is shown on the y-axis.

To further investigate the influence of FdeC on immune response in lungs during a chicken systemic infection, changes in expression of innate immune genes were determined by multiplexed qPCR (Additional file 9). This analysis revealed differential expression of distinct sets of genes compared to animals infected with the WT to animals infected with *ΔfdeC* strain after 6, 24, and 48 h (Figure 7, Additional file 10). Also, it was observed that the highest differences in the gene expression profile were particularly notable after 48 h. Among these differentially expressed genes (DEGs), six are assigned in the Panther GO database to the biological process “Inflammatory response” or “Regulation of inflammatory response” i.e., TIRAP, SELE, IL6, TLR15, CCL4 and PTGS2. Four of these genes had higher expression in lungs of chickens infected with WT strain. Moreover, it was found that TLR15 and TIRAP were also associated with the “Toll-like receptor signalling pathway” biological process in the same database. Taken together, the chicken experiments show the small difference in immune responses elicited by the APEC IMT5155 WT and its *ΔfdeC* isogenic mutant during the initial phase of infection development.

## Discussion

Autotransporters are a large family of proteins that play a central role in mediating interactions of bacteria with the environment and the host [51]. FdeC belongs to the Ve group of inverse autotransporter proteins and was previously implicated in the adhesion to host cells in the UPEC strain 536, but not the EHEC strain N39 [46, 52]. Previously, we have shown that *fdeC* transposon insertion mutant of APEC has increased adherence to chicken intestinal epithelial cells [17]. As the function of this protein in *E. coli* is not fully understood, we aimed to investigate the role of FdeC expression on adhesive properties of APEC IMT5155.

Deletion of *fdeC* did not lead to any changes in adhesion of APEC IMT5155 to chicken CHIC-8E11 cells at 37 °C, but growth of bacteria at 42 °C revealed differences between the WT and the mutant. Reporter system analyses revealed temperature dependent FdeC expression, which is in line with the study of Easton et al. [46], where FdeC expression in the EHEC strain was only detected in temperatures equal or above 39 °C. In common with FdeC, the Ag43 adhesin has been found to exhibit temperature-dependent expression, although in this case higher expression was observed at 37 °C compared to lower temperatures [53]. Although FdeC regulation in EHEC was also pH-dependent, our reporter system sensitivity did not allow for determination of whether FdeC expression in APEC is also regulated by pH of the solution. Study of FdeC in UPEC indicated that contact with host cells lead to FdeC expression [52]. These data suggest that environmental and host cues like temperature, pH and other factors are key determinants of *fdeC* regulation in UPEC, EHEC and APEC that need to be taken into consideration in future studies involving FdeC.

Our subsequent experiments revealed that expression of FdeC decreases motility in a NaCl concentration-dependent manner. Proteomic analyses showed that deletion of *fdeC* affects outer membrane ion transporters, including NhaB, a Na^+^/H^+^ antiporter [54]. NhaB expression and concentration of Na^+^ ions might lead to better control of proton motive force at 42 °C and therefore higher motility of the Δ*fdeC* strain [55]. The observed phenotype might also be explained by the decrease of sodium intracellular levels caused by NhaB expression, which subsequently can downregulate negative regulators of motility. We found that YjbN, a negative regulator of motility, has lower abundance in the Δ*fdeC* strain at 42 °C in our proteomics data, which could also explain the influence of FdeC expression on the bacterial motility [56]. Furthermore, RssAB, facilitator of RpoS degradation by the protease ClpXP was overexpressed in the Δ*fdeC* strain [57]. RpoS expression negatively regulates expression of flagellar master regulator *flhDC*, therefore its degradation facilitated by RssAB could be another factor contributing to higher motility of the Δ*fdeC* strain [58]. These changes suggest a complex regulatory network involving FdeC, reminiscent of other adhesins like YadA in *Yersinia*, which also modulates bacterial motility and microcolony formation [59]. The relationship between bacterial virulence factors such as adhesins and other phenotypic traits like hypermotility is often characterized by a complex trade-off aimed at optimizing bacterial fitness. For example, when type 1 fimbriae are highly expressed, bacterial motility is suppressed [60]. This reduced motility facilitates bacterial adherence to host surfaces rather than planktonic bacteria that may be at risk removal from the host with intestinal contents. As FdeC was previously shown to contribute to binding to surfaces, its expression might be a cue to decreased cellular motility and switch from planktonic to sessile/adhering lifestyle [46]. However, further research is needed to fully exploit the role of bacterial autotransporter adhesins in regulation of other virulence determinants in infection relevant conditions.

As expression of FdeC affects bacterial motility and the *fdeC* gene is present at a lower frequency in nonpathogenic *E. coli* strains, we hypothesised that FdeC evolution associated with pathogenic bacteria could lead to sequence variation and phenotypic divergence between APEC and nonpathogenic *E. coli* [61]*.* Consistent with this hypothesis we identified a set of FdeC variants specifically associated with APEC or nonpathogenic *E. coli*, and adhesion of the strain expressing the nonpathogenic-associated FdeC variant was lower than the strain expressing the APEC-associated variant. Influence of sequence variation on adhesin functionality was also shown for another autotransporter- Ag43, where primary structure of the protein was affecting cell aggregation kinetics and cell cluster formation [62]. As the presence of the APEC-associated FdeC variant alters expression of other virulence factors, the mode of action for different FdeC variants and its role in host colonization requires further research.

As our in vitro assays revealed greater adhesion of the Δ*fdeC* strain and a possible role of FdeC in regulation of the APEC motility, we investigated the animal infection phenotype associated with the *fdeC* deletion. We employed a chicken model of intratracheal systemic infection [63] in which we observed very low colonization of lungs, spleen and liver by both the WT and Δ*fdeC* strains. The reason of the much lower level of colonisation compared with previous reports might be explained by the major histocompatibility complex (MHC) haplotype of the chicken line used in our experiments. The AU L22 B^15^ chicken line originate from the Cornell line K and was not previously tested for APEC susceptibility, but the US ADOL line 15I with the same MHC haplotype has shown susceptibility to an APEC challenge in previous studies [41, 64]. Other differences such as the strain used for the animal challenge or the age of the animals used in the experiments may also explain the different outcome of infection. IM5155 is a well-established APEC model strain, but the reported doses used in in vivo experiments are noticeably higher than those used for APEC O1 (O1:K1:H7) [41, 65]. In contrast, the aforementioned studies used considerably younger chickens for the experiments than used in our study. Difference in lesion score or antibody production after APEC infection were observed previously, therefore it is plausible to consider them as factors that could affect the chicken colonization in our experiments [66].

Presence or absence of antigens on the surface of the bacterial envelope can lead to different immune host responses [67], therefore we aimed to establish the differences in immune response in the chicken lung following the experimental infection with the WT and Δ*fdeC* strains. The WT strain induced activation of an inflammatory responses with increased IL6 expression detected in lungs of chickens infected with the WT strain as typical for APEC infection. IL6 expression in infected lungs was previously attributed to heterophils and macrophages [68]. Stimulation of IL6 was previously associated with Toll-like receptor 4 (TLR4) stimulation by bacterial LPS, and our data is therefore consistent with the conclusion that expression of FdeC is associated with a more typical recognition of LPS by the immune system during lung colonization [69]. Differences between the WT and Δ*fdeC* strains were further characterized by changes in TLR expression. Infection of chickens with the Δ*fdeC* strain infection resulted in higher expression of TLR15, activation of which has been reported to be mediated by microbial proteases [70]. A similar outcome was observed in a *Salmonella* infection of chickens, where mutants that lack fimbrial proteins have been shown to result in an altered cytokine expression profile compared to the WT strains. Specifically, changes in the expression of IL6 and various TLRs, like TLR4 and TLR5, were observed [71]. Our data from the chicken experiments indicate that deletion of *fdeC* leads to a distinct immune response, but in vitro epithelial-, macrophage- and/or heterophile-based assays are required to provide a clearer picture of the role of FdeC in the immune response of the various cells that APEC encounters during the initial phase of lung colonization and disease development.

Overall, our findings reveal the complexity of bacterial pathogenicity and the importance of regulation of the expression of virulence factors in establishing and maintaining bacterial colonization of the host cells. The in vitro assays and systemic chicken infection model data are consistent with a supplementary role of FdeC during chicken infection. As the adhesins contribute to colonization of the gut, which is an important process for APEC carriage, future investigations should be directed towards the role of FdeC in APEC intestinal colonization and carriage.

### Supplementary Information

**Additional file 1. List of APEC genomes used in FdeC variant analysis.** Contains table with list of APEC genomes used in FdeC variant analysis in this study.

**Additional file 2. Antibodies used for flow cytometry of PBMCs.** Contains table with antibodies used for flow cytometry of PBMCs from infected chickens from the in vivo experiment.

**Additional file 3. Determination of FdeC expression inducing conditions by Western Blotting.** Contains Western blotting images.

**Additional file 4. Results from proteomics analysis.** Contains all results from proteomics analysis.

**Additional file 5. Gene set enrichment analysis for proteomics analysis.** Contains additional information for gene set enrichment analysis for proteomics analysis.

**Additional file 6. Phylogenetic tree of the FdeC variants.** Contains Unrooted phylogenetic tree based on all FdeC variants present in 10 or more isolates of APEC or nonpathogenic *E. coli*.

**Additional file 7. Role of FdeC sequence variation on adhesion of APEC IMT5155 to chicken intestinal epithelial cells.** Contains results of adhesion assays.

**Additional file 8. Colony forming unit counts for organs harvested in the chicken experiments.** Contains colony forming unit counts for organs harvested in the chicken experiments.

**Additional file 9. Results from multiplexed PCR.** Contains all results from multiplexed PCR.

**Additional file 10. Differentially expressed genes in chicken lungs infected with APEC strains.** Contains heatmap with differentially expressed genes in chicken lungs infected with APEC strains.

## Data Availability

All data generated or analysed during this study are included in this published article and its supplementary information files. The mass spectrometry proteomics data have been deposited to the ProteomeXchange Consortium via the PRIDE partner repository with the dataset identifier PXD045075.
