# Supplementary material for: FdeC expression regulates motility and adhesion of the avian pathogenic Escherichia coli strain IMT5155
Source: Vet Res. 2024 May 31;55:70. doi: 10.1186/s13567-024-01327-5 (PMC11143625; doi:10.1186/s13567-024-01327-5)
Supplement: Supplementary file 1 — Additional file 1. List of APEC genomes used in FdeC variant analysis. Contains table with list of APEC genomes used in FdeC variant analysis in this study. [file 13567_2024_1327_MOESM1_ESM.doc]

**Additional file 1 List of APEC genomes used in FdeC variant analysis.**

| BioProject number | Type of data | No of genomes listed | Succesfully downloaded |
| --- | --- | --- | --- |
| PRJNA592536 | raw | 259 | 259 |
| PRJNA488670 | assembled | 7 | 7 |
| PRJNA479542 | assembled | 97 | 95 |
| PRJNA319144 | assembled | 15 | 15 |
| PRJNA507325 | assembled | 62 | 62 |
| PRJNA553636 | raw | 32 | 32 |
| PRJEB11876 | raw | 95 | 93 |
| Single genomes* | assemblies | 10 | 10 |

* Accession numbers: CP006834, CP030791, CP030792, CP030793; CP006830.1; CP005930, CP005931, CP005932; HE962388; HE964769; AOGM00000000; AOGN00000000; AOGL00000000; CP004009; NC_008563
