# Supplementary material for: FdeC expression regulates motility and adhesion of the avian pathogenic Escherichia coli strain IMT5155
Source: Vet Res. 2024 May 31;55:70. doi: 10.1186/s13567-024-01327-5 (PMC11143625; doi:10.1186/s13567-024-01327-5)
Supplement: Supplementary file 2 — Additional file 2. Antibodies used for flow cytometry of PBMCs. Contains table with antibodies used for flow cytometry of PBMCs from infected chickens from the in vivo experiment. [file 13567_2024_1327_MOESM2_ESM.doc]

**Additional file 2 Antibodies used for flow cytometry of PBMCs from infected chickens from the in vivo experiment.**

| Abbreviation | Clone | Specificity | Fluorochrome | Panel 1 | Panel 2 |
| --- | --- | --- | --- | --- | --- |
| CD41/61-FITC^b^ | 11C3 | Chicken integrin CD41/61 | Fluorescein | Yes | No |
| Kul-01 PE^a^ | KUL01 | Chicken monocyte/macrophage | R-phycoerythrin | Yes | No |
| CD45-PerCP/Cy5.5^b,c^ | UM16-6 | Chicken CD45 | Peridinin chlorophyll-cyanine 5.5 | Yes | No |
| Bu-1-FITC^a^ | AV20 | Chicken Bu-1 | Fluorescein | No | Yes |
| TCR1-PE^a^ | TCR-1 | Chicken TCRγδ | R-phycoerythrin | No | Yes |
| CD8a-Cy5^a^ | 3-298 | Chicken CD8α | Cyanine 5 | No | Yes |
| CD4-pBlue^a^ | CT-4 | Chicken CD4 | Pacific Blue^TM^ | No | Yes |

Yes- used in the panel, No- not used in the panel

^a^ Purchased from Southern Biotech

^b^ Purchased from BIO-RAD AbD Serotec

^c^ Fluorochrome conjugation by Lightning-Link® kits from Abcam
