# Supplementary material for: FdeC expression regulates motility and adhesion of the avian pathogenic Escherichia coli strain IMT5155
Source: Vet Res. 2024 May 31;55:70. doi: 10.1186/s13567-024-01327-5 (PMC11143625; doi:10.1186/s13567-024-01327-5)
Supplement: Supplementary file 3 — Additional file 3. Determination of FdeC expression inducing conditions by Western Blotting. Contains Western blotting images. [file 13567_2024_1327_MOESM3_ESM.doc]

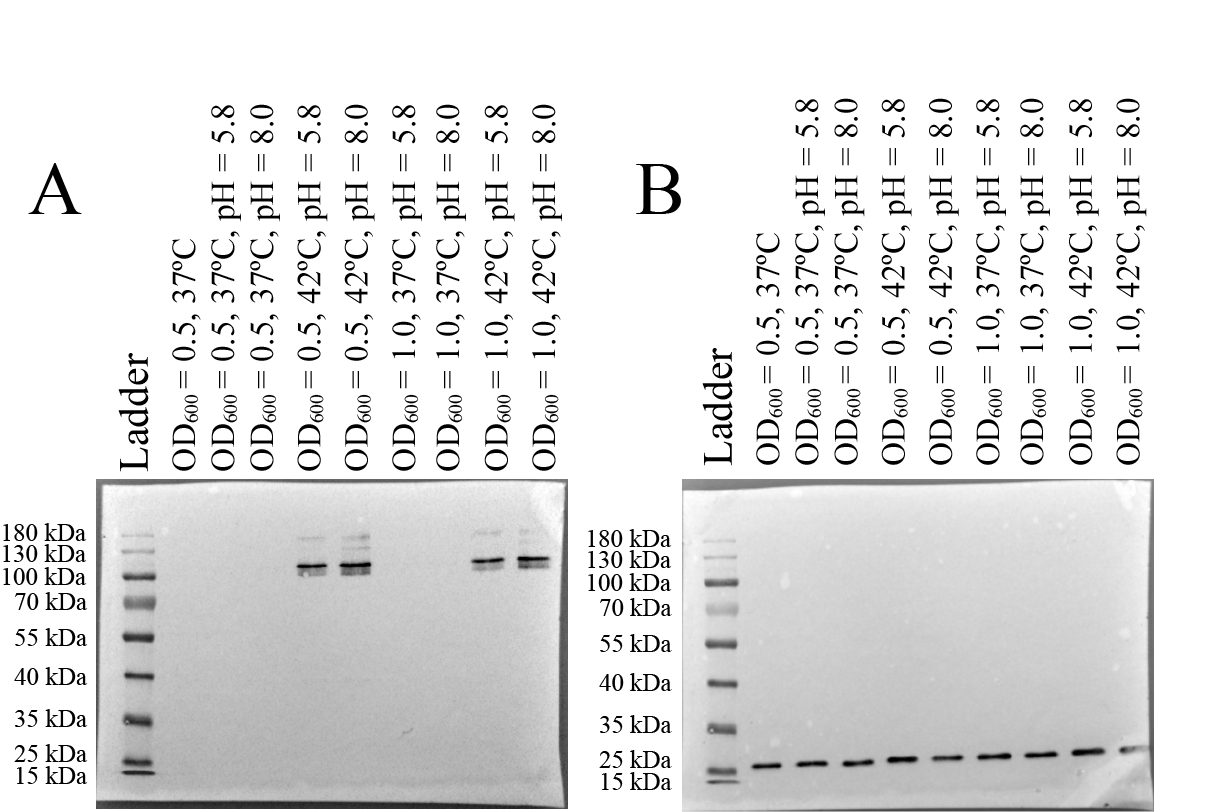


**Additional file 3 Determination of FdeC expression inducing conditions by Western Blotting.**

Ladder- protein marker (kDa), OD_600_- phase of growth (0.5, 1.0), Temperature- 37 °C or 42 °C, pH-, not adjusted (empty space), 5.8 or 8.0; A) blot developed with use of anti-HA antibody (detection of FdeC fusion protein); B) blot developed with use of anti-GFP antibody.
