# Supplementary material for: FdeC expression regulates motility and adhesion of the avian pathogenic Escherichia coli strain IMT5155
Source: Vet Res. 2024 May 31;55:70. doi: 10.1186/s13567-024-01327-5 (PMC11143625; doi:10.1186/s13567-024-01327-5)
Supplement: Supplementary file 5 — Additional file 5. Gene set enrichment analysis for proteomics analysis. Contains additional information for gene set enrichment analysis for proteomics analysis. [file 13567_2024_1327_MOESM5_ESM.doc]

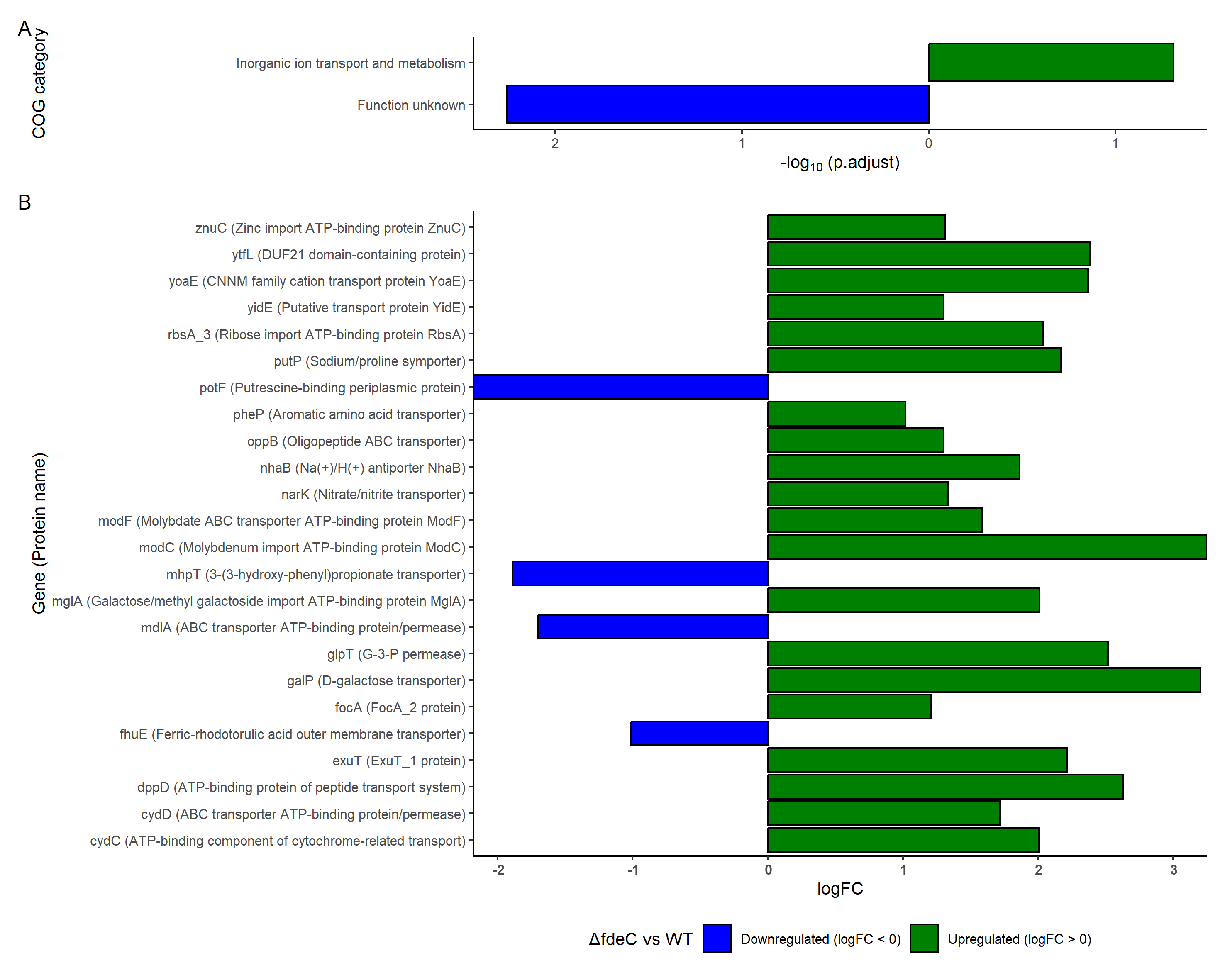


**Additional file 5. Gene set enrichment analysis for proteomics analysis.** A) Barplot showing COG categories up- or downregulated in IMT5155Δ*fdeC* strains in comparison with IMT5155 WT strain. Absolute value of logarithm of Benjamini-Hochberg corrected p value is shown on the x-axis. The COG categories are shown on the y-axis. B) Proteins significantly up- or downregulated belonging to COG category “Inorganic ion transport and metabolism”. The x-axis indicates the logFC values for each protein. Gene and protein name are shown on the y-axis.
