# Supplementary material for: FdeC expression regulates motility and adhesion of the avian pathogenic Escherichia coli strain IMT5155
Source: Vet Res. 2024 May 31;55:70. doi: 10.1186/s13567-024-01327-5 (PMC11143625; doi:10.1186/s13567-024-01327-5)
Supplement: Supplementary file 6 — Additional file 6. Phylogenetic tree of the FdeC variants. Contains Unrooted phylogenetic tree based on all FdeC variants present in 10 or more isolates of APEC or nonpathogenic E. coli. [file 13567_2024_1327_MOESM6_ESM.doc]

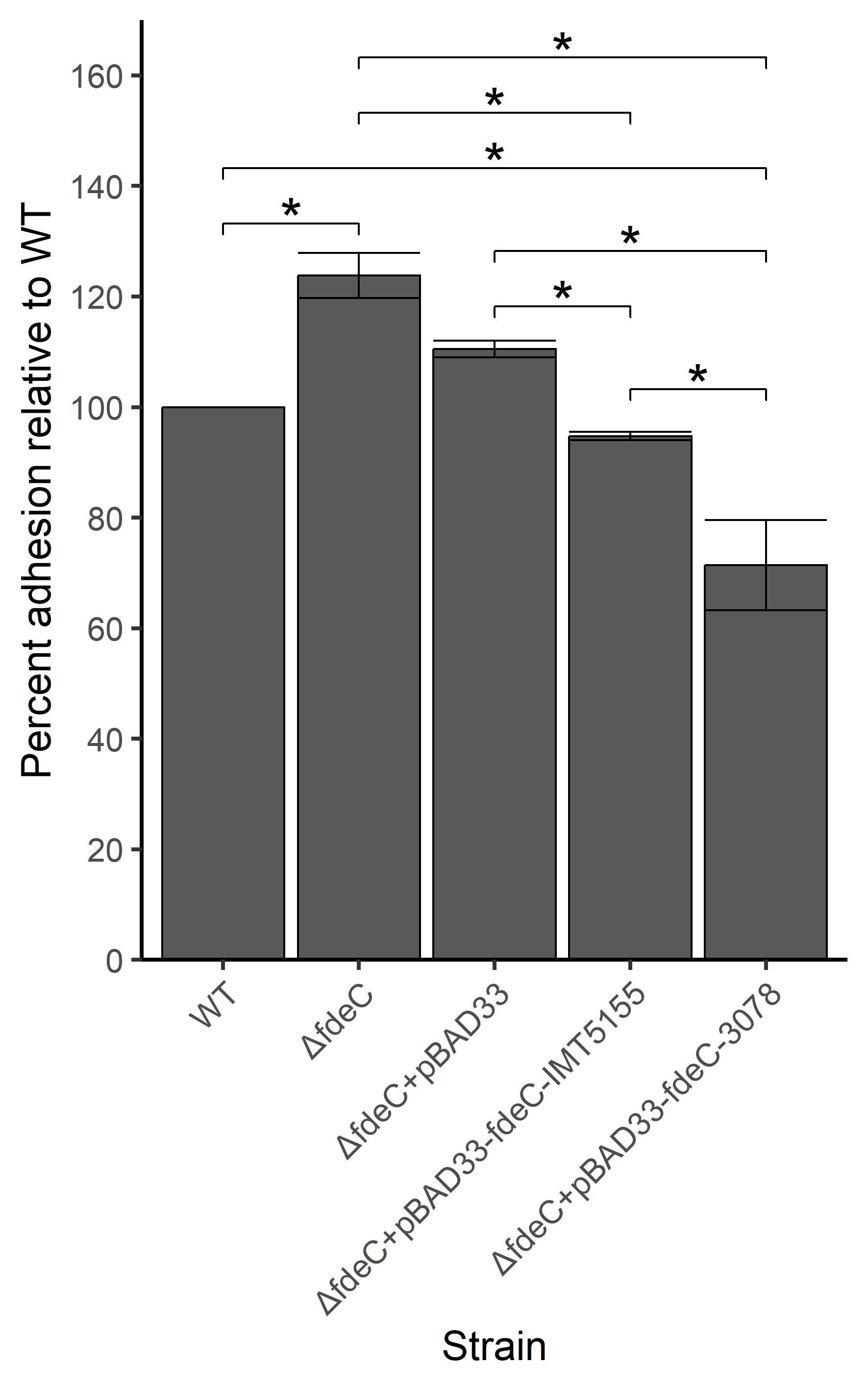


**Additional file 7 Role of FdeC sequence variation on adhesion of APEC IMT5155 to chicken intestinal epithelial cells.** Adhesion assay performed in FdeC expression inducing conditions. Names of isolates used for adhesion to CHIC-8E11 cells are shown on the x-axis. The percent of isolate’s adhesion relative to IMT5155 WT is shown on y-axis. Error bars show median absolute deviations from at least three experiments. Statistically significant observations with *p* value < 0.05 are marked with “*”. Only statistically significant comparisons are shown.
