# Supplementary material for: FdeC expression regulates motility and adhesion of the avian pathogenic Escherichia coli strain IMT5155
Source: Vet Res. 2024 May 31;55:70. doi: 10.1186/s13567-024-01327-5 (PMC11143625; doi:10.1186/s13567-024-01327-5)
Supplement: Supplementary file 7 — Additional file 7. Role of FdeC sequence variation on adhesion of APEC IMT5155 to chicken intestinal epithelial cells. Contains results of adhesion assays. [file 13567_2024_1327_MOESM7_ESM.doc]

**Additional file 8 Colony forming unit counts for organs harvested in the chicken experiments.**

| Animal | Organ | Median CFU | MAD CFU | Group | Time [h] |
| --- | --- | --- | --- | --- | --- |
| 1 | Lung | 3400 | 1482.6 | Control | 6 |
| 2 | Lung | 4000 | 296.52 | Control | 6 |
| 13 | Spleen | 1400 | 148.26 | Mutant | 6 |
| 15 | Lung | 300 | 148.26 | Mutant | 6 |
| 17 | Lung | 3300000 | 741300 | WT | 6 |
| 17 | Spleen | 3100 | 593.04 | WT | 6 |
| 21 | Spleen | 2800 | 889.56 | WT | 6 |
| 25 | Liver | 300 | 148.26 | WT | 6 |
| 25 | Lung | 45000 | 7413 | WT | 6 |
| 25 | Spleen | 2800 | 296.52 | WT | 6 |
| 38 | Spleen | 76000 | 5930.4 | Mutant | 24 |
| 43 | Spleen | 1400 | 296.52 | WT | 24 |
| 54 | Spleen | 3600 | 444.78 | Control | 48 |
| 57 | Lung | 49000 | 5930.4 | Mutant | 48 |
| 69 | Lung | 3000 | 1482.6 | WT | 48 |
| 74 | Spleen | 700 | 296.52 | WT | 48 |
