# Supplementary material for: FdeC expression regulates motility and adhesion of the avian pathogenic Escherichia coli strain IMT5155
Source: Vet Res. 2024 May 31;55:70. doi: 10.1186/s13567-024-01327-5 (PMC11143625; doi:10.1186/s13567-024-01327-5)
Supplement: Supplementary file 9 — Additional file 9. Results from multiplexed PCR. Contains all results from multiplexed PCR. [file 13567_2024_1327_MOESM9_ESM.doc]

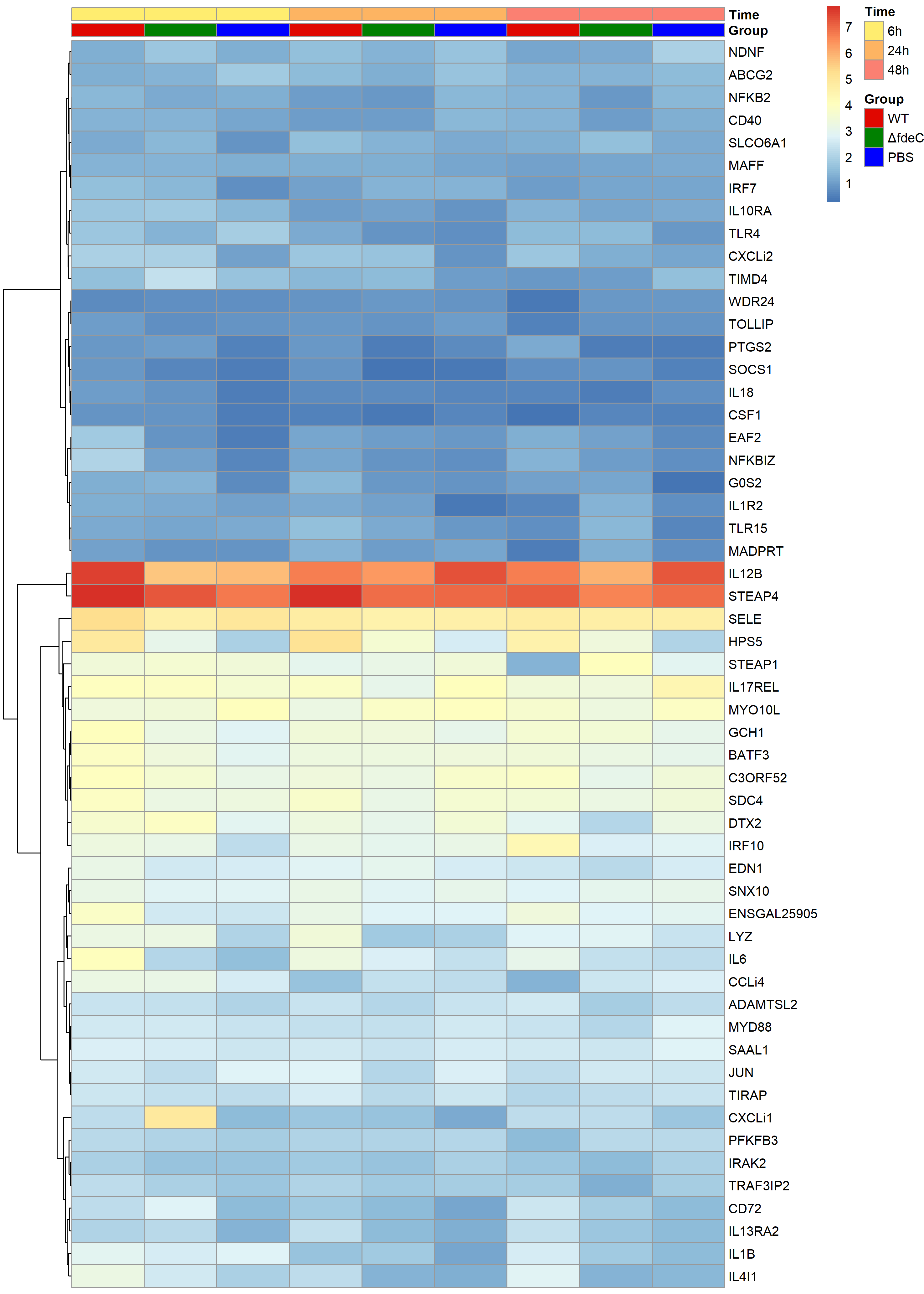


**Additional file 10. Differentially expressed genes in chicken lungs infected with APEC strains.** Heatmap with differentially expressed genes in the chicken lungs obtained 6, 24 or 48 h after intratracheal inoculation with WT, Δ*fdeC* strains or PBS. Median logFC values for each gene for each group (five chickens per groups) are presented as one rectangle. Colour gradient is proportional to the median logFC values of each gene in each group and timepoint.
